# Supplementary material for: Allosteric regulation and crystallographic fragment screening of SARS-CoV-2 NSP15 endoribonuclease
Source: Nucleic Acids Res. 2023 Apr 28;51(10):5255–70. doi: 10.1093/nar/gkad314 (PMC10250223; doi:10.1093/nar/gkad314)
Supplement: gkad314_Supplemental_Files [file gkad314_supplemental_files.zip › Supplementary datasets.zip!/159/Refine_4/01-BUSTER/Cycle-1/shell.01/R_Rfree.html]

# Rxpct factors in resolution bins at cycle 0


|  |  |  |  |  |  |  |  |  |
| --- | --- | --- | --- | --- | --- | --- | --- | --- |
|  | **<d> (Å)**  Rxpct\_Work | Rxpct\_Free | Rxpct\_All | |  |  |  |  |
|  | 12.8357 | 0.3191 | 0.3984 | 0.3225 |  |
|  | 7.4107 | 0.2993 | 0.3089 | 0.2999 |  |
|  | 5.7403 | 0.2873 | 0.2862 | 0.2872 |  |
|  | 4.8514 | 0.1866 | 0.1886 | 0.1868 |  |
|  | 4.2786 | 0.1716 | 0.1562 | 0.1707 |  |
|  | 3.8701 | 0.1903 | 0.1899 | 0.1903 |  |
|  | 3.5600 | 0.2106 | 0.2125 | 0.2107 |  |
|  | 3.3142 | 0.2176 | 0.2338 | 0.2186 |  |
|  | 3.1131 | 0.2020 | 0.2010 | 0.2020 |  |
|  | 2.9447 | 0.2033 | 0.2042 | 0.2034 |  |
|  | 2.8010 | 0.1979 | 0.1807 | 0.1971 |  |
|  | 2.6764 | 0.1917 | 0.1964 | 0.1919 |  |
|  | 2.5671 | 0.2059 | 0.1923 | 0.2051 |  |
|  | 2.4702 | 0.2092 | 0.2055 | 0.2090 |  |
|  | 2.3835 | 0.2075 | 0.2052 | 0.2073 |  |
|  | 2.3054 | 0.1995 | 0.2159 | 0.2003 |  |
|  | 2.2344 | 0.2051 | 0.2119 | 0.2054 |  |
|  | 2.1696 | 0.2037 | 0.2065 | 0.2038 |  |
|  | 2.1102 | 0.2175 | 0.2187 | 0.2176 |  |
|  | 2.0554 | 0.2457 | 0.2239 | 0.2446 |  |
|  | **Overall** | 0.2151 | 0.2166 | 0.2151 |  |

# Rxpct factors in resolution bins at cycle 1


|  |  |  |  |  |  |  |  |  |
| --- | --- | --- | --- | --- | --- | --- | --- | --- |
|  | **<d> (Å)**  Rxpct\_Work | Rxpct\_Free | Rxpct\_All | |  |  |  |  |
|  | 12.8357 | 0.3191 | 0.3964 | 0.3224 |  |
|  | 7.4107 | 0.2988 | 0.3083 | 0.2994 |  |
|  | 5.7403 | 0.2853 | 0.2824 | 0.2852 |  |
|  | 4.8514 | 0.1866 | 0.1878 | 0.1867 |  |
|  | 4.2786 | 0.1707 | 0.1586 | 0.1701 |  |
|  | 3.8701 | 0.1904 | 0.1909 | 0.1904 |  |
|  | 3.5600 | 0.2104 | 0.2093 | 0.2103 |  |
|  | 3.3142 | 0.2160 | 0.2308 | 0.2169 |  |
|  | 3.1131 | 0.2011 | 0.2002 | 0.2010 |  |
|  | 2.9447 | 0.2028 | 0.2001 | 0.2027 |  |
|  | 2.8010 | 0.1975 | 0.1834 | 0.1968 |  |
|  | 2.6764 | 0.1910 | 0.2000 | 0.1915 |  |
|  | 2.5671 | 0.2050 | 0.1927 | 0.2043 |  |
|  | 2.4702 | 0.2089 | 0.2016 | 0.2085 |  |
|  | 2.3835 | 0.2078 | 0.2072 | 0.2078 |  |
|  | 2.3054 | 0.1994 | 0.2191 | 0.2003 |  |
|  | 2.2344 | 0.2066 | 0.2162 | 0.2070 |  |
|  | 2.1696 | 0.2049 | 0.2092 | 0.2051 |  |
|  | 2.1102 | 0.2190 | 0.2158 | 0.2189 |  |
|  | 2.0554 | 0.2456 | 0.2323 | 0.2449 |  |
|  | **Overall** | 0.2146 | 0.2163 | 0.2147 |  |

# Rxpct factors in resolution bins at cycle 2


|  |  |  |  |  |  |  |  |  |
| --- | --- | --- | --- | --- | --- | --- | --- | --- |
|  | **<d> (Å)**  Rxpct\_Work | Rxpct\_Free | Rxpct\_All | |  |  |  |  |
|  | 12.8357 | 0.3253 | 0.3987 | 0.3284 |  |
|  | 7.4107 | 0.3015 | 0.3133 | 0.3022 |  |
|  | 5.7403 | 0.2836 | 0.2927 | 0.2839 |  |
|  | 4.8514 | 0.1887 | 0.1902 | 0.1888 |  |
|  | 4.2786 | 0.1730 | 0.1627 | 0.1725 |  |
|  | 3.8701 | 0.1914 | 0.1900 | 0.1913 |  |
|  | 3.5600 | 0.2105 | 0.2074 | 0.2103 |  |
|  | 3.3142 | 0.2166 | 0.2368 | 0.2178 |  |
|  | 3.1131 | 0.2041 | 0.2093 | 0.2043 |  |
|  | 2.9447 | 0.2031 | 0.2020 | 0.2031 |  |
|  | 2.8010 | 0.1993 | 0.1878 | 0.1987 |  |
|  | 2.6764 | 0.1923 | 0.1886 | 0.1921 |  |
|  | 2.5671 | 0.2022 | 0.1933 | 0.2016 |  |
|  | 2.4702 | 0.2043 | 0.1990 | 0.2041 |  |
|  | 2.3835 | 0.2047 | 0.2021 | 0.2046 |  |
|  | 2.3054 | 0.1956 | 0.2138 | 0.1965 |  |
|  | 2.2344 | 0.2026 | 0.2090 | 0.2029 |  |
|  | 2.1696 | 0.2048 | 0.2070 | 0.2049 |  |
|  | 2.1102 | 0.2181 | 0.2148 | 0.2179 |  |
|  | 2.0554 | 0.2444 | 0.2246 | 0.2434 |  |
|  | **Overall** | 0.2153 | 0.2178 | 0.2154 |  |

# Rxpct factors in resolution bins at cycle 3


|  |  |  |  |  |  |  |  |  |
| --- | --- | --- | --- | --- | --- | --- | --- | --- |
|  | **<d> (Å)**  Rxpct\_Work | Rxpct\_Free | Rxpct\_All | |  |  |  |  |
|  | 12.8357 | 0.3134 | 0.3755 | 0.3160 |  |
|  | 7.4107 | 0.2772 | 0.2997 | 0.2786 |  |
|  | 5.7403 | 0.2609 | 0.2813 | 0.2617 |  |
|  | 4.8514 | 0.1850 | 0.1983 | 0.1857 |  |
|  | 4.2786 | 0.1697 | 0.1666 | 0.1695 |  |
|  | 3.8701 | 0.1900 | 0.2039 | 0.1907 |  |
|  | 3.5600 | 0.2043 | 0.2191 | 0.2051 |  |
|  | 3.3142 | 0.2094 | 0.2319 | 0.2107 |  |
|  | 3.1131 | 0.1989 | 0.2102 | 0.1994 |  |
|  | 2.9447 | 0.1962 | 0.2088 | 0.1967 |  |
|  | 2.8010 | 0.1898 | 0.1835 | 0.1895 |  |
|  | 2.6764 | 0.1849 | 0.1966 | 0.1855 |  |
|  | 2.5671 | 0.1933 | 0.1952 | 0.1934 |  |
|  | 2.4702 | 0.1965 | 0.1958 | 0.1965 |  |
|  | 2.3835 | 0.1945 | 0.2004 | 0.1947 |  |
|  | 2.3054 | 0.1872 | 0.2097 | 0.1883 |  |
|  | 2.2344 | 0.1949 | 0.2122 | 0.1956 |  |
|  | 2.1696 | 0.1973 | 0.2030 | 0.1976 |  |
|  | 2.1102 | 0.2125 | 0.2052 | 0.2122 |  |
|  | 2.0554 | 0.2359 | 0.2225 | 0.2352 |  |
|  | **Overall** | 0.2071 | 0.2186 | 0.2077 |  |
